# The Book World of Medicine and Science

**Published:** 1902-06-07

**Authors:** 


### THE BOOK WORLD OF MEDICINE AND SCIENCE.

THE ARTIFICIAL FEEDING OF INFANTS. By W. B. CHEADLE, M.D. Fifth edition. Edited and revised by F. J. POYNTON, M.D. Pages 268. (London: Smith Elder & Co. 1902. Price 5s.)

DR. CHEADLE'S little book on the artificial feeding of children is too well known to require detailed description, and the present volume, though revised and enlarged, presents so few alterations in the general scheme and in the text that only a most careful comparison with former editions will reveal the alterations and additions effected in it by the new editor, Dr. F. J. Poynton. The corrections which have been made refer for the most part to the chemical composition of various kinds of prepared milk and artificial foods, and the new matter which has been introduced concerns mainly the preparation and applications of graduated milk—a method which is now almost universally adopted in America, and which is slowly but surely gaining adherents in this country. In Chapter V. some space is occupied by the consideration of what Dr. Cheadle calls acholia. This condition is a very frequent one among children between two and ten years of age, although it does not generally find an independent place among the dietetic diseases of children. This condition is practically the same as that which is described by Dr. Gee as cœliac disease, and is possibly identical with the group of symptoms which Dr. Eustace Smith calls mucous disease. The diagnostic symptoms of these diseases, whether identical or different, are the frequent passing of colourless, greasy, offensive stools, with general malnutrition and wasting. The pathology of this morbid state has as many explanations as the disease has names. Dr. Cheadle inclines, however, to the belief that the pancreas, or possibly the pancreas and liver are at fault, and this latter explanation is, we believe, the true one. The treatment recommended by the author is to regulate the diet in such a way as to spare the work of these two organs, and with this object in view he enjoins restriction of starches, sugars, and fats, with an occasional dose of grey powder. With these precautions Dr. Cheadle believes that a cure will generally be effected in a few weeks. We cannot help thinking, however, that in face of the usually inveterate and stubborn course of the disease this is rather an optimistic prognosis. The paragraphs which are devoted to the discussion of dilatation of the stomach and the disorders to which it may give rise will be thoroughly appreciated by those who take practical interest in the treatment of children. The gastric capacity, according to Fleischmann, is greater at the same age in artificially-fed infants than in those who are breast-reared; and this consequence is undoubtedly due to faulty feeding. This increase of capacity noticed by Fleischmann is only a minor degree of that mentioned in Dr. Cheadle's work as referable to over-feeding and conducive to constant vomiting, overflow, flatulent distension, malnutrition and wasting; in fact to some of the most frequent disorders of childhood. Having had in the case of former editions the privilege of reviewing Dr. Cheadle's book, and having been denied on those occasions that legitimate *solacium* of the reviewer, namely a just opening for criticism of fact, we cannot resist on the present occasion pointing out a very glaring error of arithmetic which appears on page 106. It is there stated that a deficiency of $1\frac{1}{2}$ per cent. of fat in 2 ozs. of milk mixture can be made good by the addition of about five drops of cream. According to our reckoning instead of five drops something over seventy drops of cream must be added to make the percentage correct. When Dr. Cheadle's book first saw light in 1889 it undoubtedly held the first position among works on the artificial feeding of children, and with the additions and corrections which have been introduced in subsequent editions there is no reason to suppose that at the present time it has in any way lost this position. Entirely free from prejudice of all kinds, and holding no brief for any particular system of feeding, it stands out prominently among other works of the same kind as a thoroughly practical guide for those who have the dietetic management of children.

SCIENTIFIC RESEARCH. A View from Within. By STEPHEN SMITH, M.R.C.S. (London: Elliot Stock. 1901. Price 2s.)

THIS is a highly-coloured anti-vivisectionist account of the methods of the physiologists. The author has taken certain experiments which he says he has seen done, and then, having repeated them himself on dead animals, has drawn pictures thereof, which, being reproduced in colours, form the basis of this work. Of course the intention is to discredit the physiologists, but an author publishing such a book as this in England ought surely to know that much that he describes could not occur in England under the present restrictive laws.

PHOTOGRAPHIC ATLAS OF THE DISEASES OF THE SKIN. By GEORGE HENRY FOX, A.M., M.D. (Philadelphia and London: J. B. Lippincott Company).

THE final fasciculi of these admirable plates confirm us in the favourable opinion we have already expressed regarding them. Nothing could be more true to life than the representation of the wheals of urticaria in one of the pictures of that disease, while the plate illustrating dermatitis exfoliativa serves well to show how accurately the method of reproduction adopted is capable of representing morbid conditions. We would also again speak favourably of the accompanying letterpress, which forms a very useful and practical treatise on cutaneous therapeutics.

